# Exclusive breastfeeding practices in an urban settlement of Vellore, southern India: findings from the MAL-ED birth cohort

**DOI:** 10.1186/s13006-019-0222-0

**Published:** 2019-06-27

**Authors:** Samarasimha Reddy N., Kulandaipalayam Natarajan Sindhu, Karthikeyan Ramanujam, Anuradha Bose, Gagandeep Kang, Venkata Raghava Mohan

**Affiliations:** 1 The Wellcome Trust Research Laboratory, Division of Gastrointestinal Sciences, Christian Medical College, Vellore, Tamil Nadu 632004 India; 2 Department of Community Health, Christian Medical College, Vellore, Tamil Nadu 632002 India

**Keywords:** Exclusive breastfeeding, Indian children, Urban slums, Complementary feeding

## Abstract

**Background:**

Exclusive breastfeeding is recommended in the first six months of life. Observing breastfeeding practices and further the introduction of complementary food using a birth cohort can provide a better understanding with reference to the child’s growth and nutrition. We aim to describe the exclusive breastfeeding practices in the Indian MAL-ED birth cohort.

**Methods:**

The Indian MAL-ED (Etiology, Risk Factors, and Interactions of Enteric Infections and Malnutrition and the Consequences for Child Health) birth cohort comprises of eight contiguous urban slums in Vellore. Of the 251 children enrolled in the cohort at birth, a 24 month follow-up was completed for 228 children and data collection was from March 2010 through February 2012. Trained field research assistants collected data on exclusive breastfeeding and complementary feeding practices from birth using a structured questionnaire through a biweekly surveillance. Survival and Cox proportional hazard regression analyses were used to estimate the duration of exclusive breastfeeding and factors influencing the same.

**Results:**

Breastfeeding was initiated within the first hour of birth in 148 (59%) infants. Colostrum was given in 225 (89.6%) infants whilst 32 (12.7%) infants received prelacteal feeds. Exclusive breastfeeding up to four months was observed in 55 (22.1%, 95% Confidence Interval [CI] 17.1%, 27.5%) infants with only three (1.1%, 95% CI 0.2%, 3.5%) of the cohort mothers continuing to exclusively breastfeed up to six months. Cox proportional hazard regression analysis revealed no gender differences to being exclusive breastfed (Adjusted Hazard Ratio [AHR] 0.97; 95% CI 0.74, 1.27). Children from families of low socioeconomic status had a lower risk of early cessation of exclusive breastfeeding compared to children from middle or higher socioeconomic status (AHR 0.52; 95% CI 0.38, 0.71).

**Conclusions:**

Early initiation of exclusive breastfeeding is important and improving rates suggest continuation of efforts in this direction energetically. Continuation of exclusive breastfeeding practice is significantly low in these urban slums with introduction of animal milk and complementary foods even before six months of age. This highlights the urgent need to evaluate pragmatic interventions to raise awareness on the importance of exclusive breastfeeding and its practice.

## Background

Exclusive breastfeeding (EBF) means the infant receives only breast milk and no other liquids or solids, not even water with the exception of oral rehydration solution (ORS), syrup or drops of vitamins, minerals, or medicines [1]. The current recommendation by World Health Organization (WHO) is exclusive breastfeeding for six months duration, and further, to initiate complementary foods at six months of age while the mother continues to concurrently breastfeed her baby up to 24 months of age [2]. Exclusive breastfeeding has many proven benefits to both the infant and mother. Breast milk contains all the required nutrients during first six months of life, protects against respiratory infections and gastrointestinal infections; and further reduces the risk of being overweight and obese in childhood and adolescence [3–6]. Exclusive breastfeeding has also been associated with a higher intelligent quotient (IQ) in children [7]. Studies have shown indirect benefits on mother’s health by protecting against breast and ovarian cancer [8]. There is also evidence that breastfeeding reduces the risk of type-2 diabetes [8]. Exclusive breastfeeding delays the return of menstruation and fertility after childbirth and can importantly contribute to spacing of childbirths [8].

In low and middle income countries, only 37% of the infants are exclusively breastfed up to six months of age [7]. In India, as per National Family Health Survey-4 (NFHS-4), 54.9% of infants are exclusively breastfed up to six months of age [9]. A wide range of factors interplay, influencing the mother’s decision to exclusively breastfeed her baby, and to continue breastfeeding, until her infant is two years of age [10, 11]. These factors include a complex array of cultural and social factors; peer behaviours and pressures, and the availability of healthcare services [10, 11]. Often breastfeeding and weaning practices in developing settings are from cross-sectional studies and these estimates could potentially be blurred because of the element of recall bias. The primary purpose of this study is to describe the exclusive breastfeeding practices in the Indian MAL-ED birth cohort during the first six months of life and to assess the socioeconomic factors influencing the duration of exclusive breastfeeding.

## Methods

The Fogarty International Centre of the National Institutes of Health and the Foundation for the National Institutes of Health led the MAL-ED (The Etiology, Risk Factors, and Interactions of Enteric Infections and Malnutrition and the Consequences for Child Health) study, a multi-national birth cohort study that was established at eight countries. Christian Medical College established the Indian birth cohort at Vellore, a city situated 120 km from Chennai, the state capital of Tamil Nadu in south India. This cohort residing in eight contiguous urban settlements of Vellore old town were followed-up from March 2010 through February 2012. In the year 2012, these eight urban settlements had a population estimate of around 13,000 with an average family size of 5.7 (3–13), population density of around 42,000 per km^2^ and an infant mortality rate of 38 per 1000 live births [12]. A child born as a singleton, parent/primary caregiver of the child being a permanent resident of the study area and those willing to permit home visits by the designated field research assistant were included in the study. Parents/primary caregivers of the child who were likely to be away from the study site for more than 30 days during the study, new-borns of teenage mothers, prolonged hospitalization of the neonate at birth, diagnosed with a chronic condition or enteropathy and those who weighed less than 1500 g at the time of enrolment were excluded from the study [13]. A written informed consent was obtained from the mother/primary caregiver of the study participant.

### Data collection and analysis

**Fig. 1:**
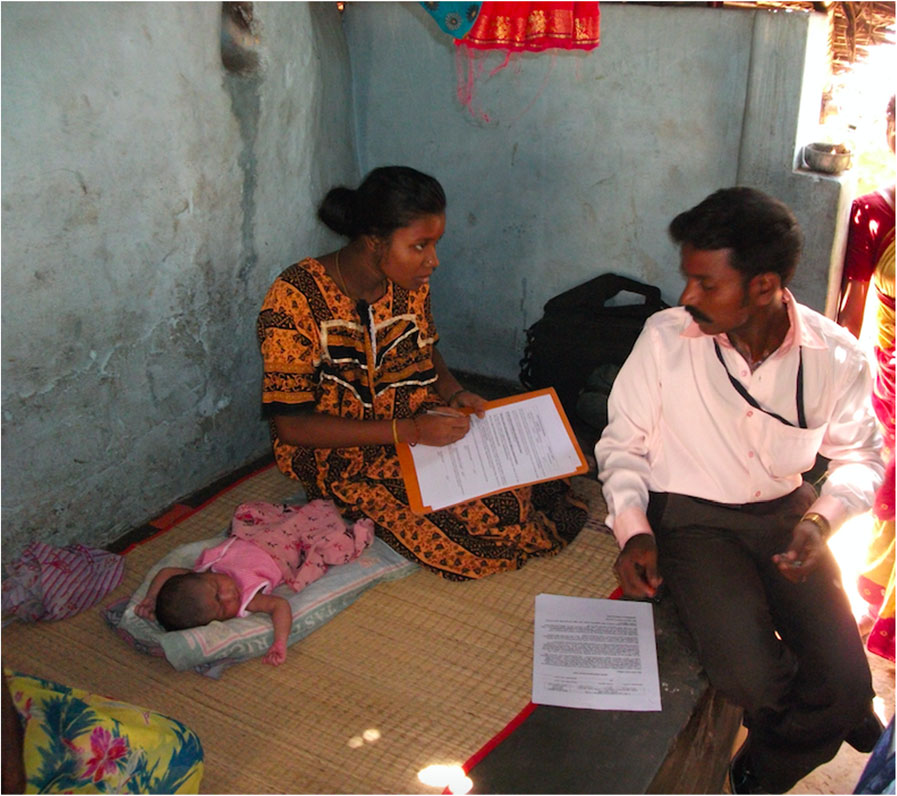
Field research assistant interviewing mother about feeding practices

Trained field research assistants collected data on breastfeeding and complementary foods introduced using a structured, validated questionnaire through an intensive bi-weekly surveillance through home visits (Fig. 1) [14]. The data collected included the baseline sociodemographic characteristics, time of initiation of breastfeeding, period of exclusive breastfeeding, continued breastfeeding following introduction of complementary foods and type of complementary foods introduced (Fig. 1) [14]. *“Exclusive breastfeeding”* was defined as breastfeeding alone with no other liquids or solids from birth. Socioeconomic status (SES) was evaluated using the WAMI index (access to improved Water and sanitation, eight selected Assets, Maternal education and monthly household Income), an index developed to measure SES across diverse settings of low and middle income countries. The final computed score further led to the stratification of SES into low, middle and high using tertiles of the overall score [15]. Data analyses were performed using STATA 13 software (StataCorp, College Station, TX, USA). A descriptive analysis followed by a Kaplan Meier survival analysis was used to estimate the duration of exclusive breastfeeding and introduction of complementary foods during the first six months of life. Socioeconomic factors influencing the duration of exclusive breastfeeding practices during the first six months was studied using Cox proportional hazards regression analysis with the dependent variable being age in days at which EBF was stopped. The independent sociodemographic variables such as gender, birthweight, mother’s age, and socioeconomic status were selected to fit the model. For survival analysis and Cox proportional hazard analysis, children were censored on the day on which they were lost to follow-up or the last valid interview was missed.

## Results

Of the 301 pregnant women (in the third trimester) who consented to participate in the study, 251 infants were enrolled in the study following delivery. A 24 month follow up was completed by 228 (90.9%) children with 23 (9.1%) children accounting for lost-to-follow, of whom 15 (65.2%) had migrated from the locality. The cohort comprised of 138 (55%) males and 113 (45%) females with 5 (2%) and 35 (14%) babies being born with very low birthweight (< 1.99 kg) and low birthweight (2–2.49 kg) respectively. Mothers of 103 (41%) infants were aged less than 23 years at the time of enrolment. Families from low socioeconomic status and middle/high socioeconomic status were 71 (31%) and 161 (61%) respectively (Table 3). The mean (SD) and median (IQR) of mothers age was 23.88 (4.09) years and 23 (21–26) years respectively.

Breastfeeding was initiated within the first hour of birth and within 24 h in 148 (59%) and 92 (36.6%) infants respectively. Colostrum was given in 225 (89.6%) infants whereas 32 (12.7%) infants received prelacteal feeds. Of the 32 infants who received prelacteal feeds, 28 received both prelacteal feeds and colostrum (Table 1). The most common prelacteal feeds used comprised of honey or vasambu (*Acorus calamus)*, an herb that is burnt and rubbed over stone to be mixed with water or breast milk and fed to the baby. The mean (SD) and median (IQR) duration of EBF were 76.9 (43.8) days and 81 (40–111) days respectively. The mean age at which the infants were started on formula feeds or animal milk were 162.3 (122.4) days and median (IQR) of 142 (68–219) days. The mean age at which clear liquids (including water) and solid foods were introduced were 117.4 (79.4) days and 122.9 (30.8) days respectively. The mean (SD) and median (IQR) duration of overall breastfeeding duration in the cohort were 468.5 (167.9) days and 481 (371–607) days respectively (Table 2).

**Table 1:** Breastfeeding practices following birth in the MAL-ED birth cohort (*N* = 251)

Breastfeeding following birth		*n* (%)
Breastfed following birth	Within one hour of birth	148 (59)
	Within 1–24 h of birth	92 (36.6)
	Within 1–3 days of birth	11 (4.4)
Feeds following birth	Colostrum	225 (89.6)
	Prelacteal feeds	32^a^(12.7)

^a^28 Infants were given both colostrum and prelacteal feeds

**Table 2:** Duration of breastfeeding in the MAL-ED birth cohort

Variable	*n*	Mean (days) ± SD	Median (days) (IQR)	Range
Exclusive breastfeeding	250	76.9 ± 43.8	81 (40–111)	1–188
Age at which first formula or animal milk was given	236	162.3 ± 122.4	142 (68–219)	5–686
Age at which first clear liquid was given (including water)	191	117.4 ± 79.4	102 (69–151)	9–456
Age at which first solid food was given	239	122.9 ± 30.8	122 (101–140)	41–216
Age at which breastfeeding was stopped	175	468.5 ± 167.9	481 (371–607)	7–731

**Fig. 2:**
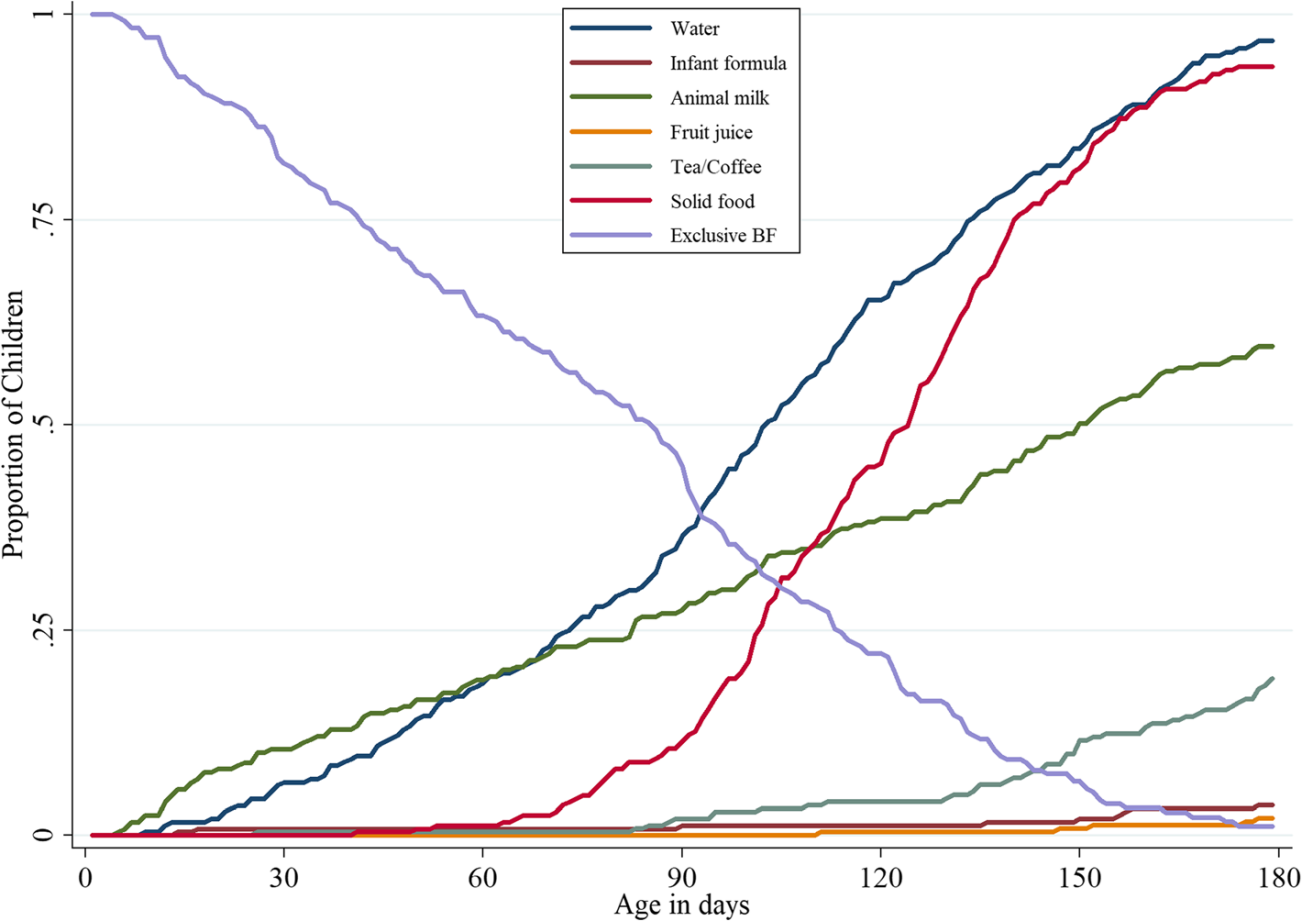
Exclusive breastfeeding and introduction of other foods in first six months of life (*N* = 251)

Exclusive breastfeeding up to four months was observed in 55 infants (22.1%; 95% CI 17.1%, 27.5%) of the cohort, with only 3 (1.1%; 95% CI 0.2, 3.5%) of the cohort mothers continuing to exclusively breastfeed up to six months of age. Animal milk (predominantly cow’s milk) was initiated as early as the first week of life in 4 (1%) infants, and 146 (58%) infants were on animal milk by six months of age. Solid or semi-solid foods were started as early as two months with 225 (90%) infants being on the same by six months of age. The semi-solid/solid foods given were porridges made from rice/wheat and mashed potatoes/banana. The use of formula milk at six months of age was among 10 (5%) infants in this cohort (Fig. 2). Cox proportional hazard regression model showed no significant differences between male and female infants in terms of duration of exclusively breastfeeding practices followed (AHR 0.97; 95% CI 0.74, 1.27). Children from low socioeconomic status families had AHR of 0.52 (95% CI 0.38, 0.71) indicating that these children had 48% lower risk of cessation of EBF compared to children from middle or higher socioeconomic status families at a significant level (Table 3).

**Table 3:** Baseline sociodemographic characteristics and factors influencing exclusive breastfeeding practices using Cox proportional hazards regression model

Variable	Category	Frequency *n* (%)	Unadjusted Hazard ratio (95% CI)	Adjusted Hazard ratio (95% CI)
Gender *(n =* 251*)*	Male	138 (55)	–	–
	Female	113 (45)	0.89 (0.69, 1.15)	0.97 (0.74,1.27)
Birthweight (kg) *(n =* 245)	Very low birthweight (< 1.99 kg)	5 (2)	1.91 (0.78, 4.67)	1.87 (0.76,4.60)
	Low birthweight (2–2.49 kg)	35 (14)	1.26 (0.87, 1.81)	1.20 (0.82,1.76)
	Normal birthweight (≥ 2.5 kg)	205 (84)	–	–
Parity of the mother (*n =* 249*)*	> 2	100 (40)	1.11 (0.85, 1.44)	0.98 (0.73,1.32)
	≤ 2	149 (60)	–	–
Age of the mother *(n =* 249*)*	< 23 years	103 (41)	–	–
	≥ 23 years	146 (59)	0.98 (0.75, 1.27)	0.94 (0.70,1.25)
Socioeconomic status (WAMI^a^) (*n* = 232)	Low (≤ 33rd centile)	71 (31)	0.56* (0.42,0.76)	0.52*(0.38,0.71)
	Middle and High (> 33rd centile)	161 (69)	–	–

*p value < 0.05

^a^WAMI is a socioeconomic index that integrates 4 components namely, access to improved water and sanitation, eight selected assets, maternal education and household income

## Discussion

In our cohort, 59% of the infants were breastfed within the first hour of birth, and this is similar to the estimates from other urban regions of Tamil Nadu with 55.4% of children being breastfed within first hour of life [16]. However this estimated proportion in our cohort is high when compared to studies done in other rural areas of south India where only 35% of the infants were breastfed within the first hour of birth [17]. Given the prevalence of traditional beliefs and cultural practices in India such as giving honey, vasambu etc., colostrum initiation in 90% of this cohort is a positive as well as an encouraging finding. The practice of prelacteal feeding was observed in 12% of our cohort which is low when compared to 33.5% from a study done in rural areas of south India [17]. This difference can be attributed to the impact of awareness programmes through the outreach services by the government as well as Christian Medical College, Vellore in the last few decades. We need to continue to encourage, reinforce, reiterate the importance of early and exclusive breastfeeding.

However, a few findings in our cohort are worrying. The mean duration of exclusive breastfeeding was 76 days, and this is less than three months. Exclusive breastfeeding for four months and six months in this cohort was followed by 22 and 1% of mothers respectively and this is very low when compared to the other urban areas of Tamil Nadu state, with NFHS-4 estimates showing EBF for six months being practiced by 47.8% of the mothers [16]. A pooled data analysis from three birth cohort studies conducted between 2002 and 2009 in Vellore has shown the prevalence of EBF at six months to be 11.4% which is much higher than our study estimate [18]. The reasons for this large difference in EBF practices might be because the data collected in the MAL-ED cohort was more rigorous with twice-a-week follow-up through home visits, whereas NFHS-4 survey being a cross-sectional survey is more prone for recall bias [19]. It is a good sign to find no gender differences in this cohort in terms of exclusive breastfeeding. Children of low socioeconomic status were at lower risk of cessation of EBF in first six months compared to their counterparts at a significant level. The possible reasons for this could be the lack of purchasing capacity of animal milk/infant formula by families of lower socioeconomic status.

This being a cohort study is the biggest strength of the study, as precise estimates of breastfeeding practices followed were collected by a bi-weekly surveillance that is not possible with cross-sectional studies with a single recall. With the finding that less than 25% of the mothers in this Indian birth cohort exclusively breastfed their babies for four months, there is a need to explore the reasons for failure in complying to the recommended EBF practice for six months. With some high income countries considering the revision of EBF guidelines from six months to four months, there is a need to understand the effects of EBF for four months when compared to six months on growth and infections, especially in low and middle income countries [20]. An important limitation in the study that could impact breastfeeding practices was that employment status of the mother was not captured.

## Conclusions

Early initiation of exclusive breastfeeding is important and improving rates suggest continuation of efforts in this direction energetically. Continuation of exclusive breastfeeding practice is significantly low in these urban slums, with introduction of animal milk and complementary foods even before six months of age. There is a need to evaluate interventions targeted to induct and escalate awareness on importance of exclusive breastfeeding among mothers and their families. A more meticulous data collection for estimating the exclusive breastfeeding practices during the next round of the national family health survey could be pivotal in providing a clear understanding of practices across country, thereby facilitating the planning of interventions, strategies, and policies.

## Data Availability

The anonymised datasets generated will be shared on request.

## Abbreviations

EBF: Exclusive Breastfeeding
IQ: Intelligent Quotient
MAL-ED: The Etiology, Risk Factors, and Interactions of Enteric Infections and Malnutrition and the Consequences for Child Health
NFHS-4: National Family Health survey 4
WHO: World Health Organization
